# Vitamin D Status Among Children With Juvenile Idiopathic Arthritis: A Multicenter Prospective, Non-randomized, Comparative Study

**DOI:** 10.3389/fped.2022.915943

**Published:** 2022-07-26

**Authors:** Elena I. Kondratyeva, Nuriniso D. Odinaeva, Leonid Ya Klimov, Nadeshda S. Podchernyaeva, Natalya I. Ilenkova, Svetlana V. Dolbnya, Elena K. Zhekaite, Victoria A. Kuryaninova, Yuliya V. Kotova, Margarita I. Tikhaya, Elena P. Shitkovskaya, Liubov V. Bychina, Tamara G. Drepa, Aisa E. Zodbinova, Yuliya L. Melyanovskaya, Nika V. Petrova, Elena V. Loshkova, Sergei I. Kutsev

**Affiliations:** ^1^Research Centre for Medical Genetics, Moscow, Russia; ^2^Research Clinical Institute for Childhood of the Moscow Region, Moscow, Russia; ^3^Stavropol State Medical University, Stavropol, Russia; ^4^Sechenov University, Moscow, Russia; ^5^Krasnoyarsk State Medical University Named After Professor V. F. Voino-Yasenetsky, Krasnoyarsk, Russia

**Keywords:** vitamin D deficiency, juvenile idiopathic arthritis, seasons of the year, genotypes, *VDR gene*

## Abstract

**Background:**

Juvenile idiopathic arthritis (JIA) is a chronic autoimmune disease characterized by destructive and inflammatory damage to the joints. The aim in this study was to compare vitamin D levels between children and adolescents, 1–18 years of age, with juvenile idiopathic arthritis (JIA) and a health control group of peers. We considered effects of endogenous, exogenous, and genetic factors on measured differences in vitamin D levels among children with JIA.

**Methods:**

Our findings are based on a study sample of 150 patients with various variants of JIA and 277 healthy children. The blood level of vitamin D was assessed by calcidiol level. The following factors were included in our analysis: age and sex; level of insolation in three regions of country (center, south, north); assessment of dietary intake of vitamin D; effect of prophylactic doses of cholecalciferol; a relationship between the TaqI, FokI, and BsmI polymorphisms of the *VDR* gene and serum 25(OH)D concentration.

**Results:**

We identified a high frequency of low vitamin D among children with JIA, prevalence of 66%, with the medial level of vitamin D being within the range of “insufficient” vitamin D. We also show that the dietary intake of vitamin D by children with JIA is well below expected norms, and that prophylactic doses of vitamin D supplementation (cholecalciferol) at a dose of 500–1,000 IU/day and 1,500–2,000 IU/day do not meet the vitamin D needs of children with JIA. Of importance, we show that vitamin D levels among children with JIA are not affected by clinical therapies to manage the disease nor by the present of VDR genetic variants.

**Conclusion:**

Prophylactic administration of cholecalciferol and season of year play a determining role in the development of vitamin D deficiency and insufficiency.

## Introduction

Juvenile idiopathic arthritis (JIA) is one of the most common rheumatic diseases. JIA is defined as arthritis of unknown etiology with a duration of >6 weeks, developing in children aged <18 years, and for a clinical diagnosis, arthritis with known etiologies or all other joint pathologies should be excluded (1, 2). The prevalence of chronic arthritis ranges from 0.07 to 4.01 per 1,000 children, with an annual incidence of 0.008– to 0.226 per 1,000 children (1, 3). In the Russian Federation, the prevalence of JIA is 62.3 per 100,000 children (2). Although the etiology of JIA is unknown but, it is likely multifactorial in nature, with a commonly suggested pathway involving pathological immune responses to various trigger factors, a commonly suggested pathway (1, 4).

Currently, it is known that the active metabolite of vitamin D, calcitriol (1,25(OH)2D), is a pleiotropic hormone that performs several important biological functions (5–7). One of the most important functions is immunomodulation, exerting regulatory effects on the proliferation, differentiation, and function of various immune cells (5, 6). Vitamin D reduces the maturation of dendritic cells and the number of Th1 and Th17 cells as well as their ability to secrete IFN-γ, IL-2, and IL-17. These actions limit the attraction and proliferation of T cells. Further, suppression of IL-12 stimulates the development of Th2 cells and their production of IL-4, IL-5, and IL-13, additionally suppressing Th1 cells and shifting the balance to a predominance of Th2 cells. Calcitriol also induces the differentiation of Treg cells that produce the regulatory cytokine IL-10, which itself suppresses the development of Th1 and Th17 cells, leading to immune tolerance. Increasing immune tolerance is crucial for limiting and maintaining an effective immune response. Resultantly, vitamin D deficiency is associated with an increased risk of autoinflammatory pathology (7–9).

Previous studies have reported an association between low vitamin D content, measured as the serum calcidiol (25(OH)D) level, and higher frequency and severity of autoimmune diseases, such as multiple sclerosis, type 1 diabetes mellitus, inflammatory bowel diseases, and systemic lupus erythematosus (5, 10–16). The association between vitamin D and JIA is similarly being actively studied worldwide (1, 17, 18). However, the effect of vitamin D levels on the development and disease activity of JIA remains unclear. Therefore, this study aimed to compare vitamin D availability, measured as the serum calcidiol level, between patients with JIA and their healthy peers, controlling for endogenous and exogenous factors. As part of a multicenter study, the frequency of vitamin D deficiency in healthy children and patients with JIA at different age periods in three regions of the Russian Federation (center, north, and south) was examined. These regions result in different levels of insolation; in each of the four seasons of the year. To the best of our knowledge, this topic, in conjunction with the vitamin D intake through diet and in consideration of genetic factors, was studied for the first time.

## Materials and Methods

### Type and Design of the Study and Statement of Ethics

This multicenter, prospective, comparative, open, non-randomized study was conducted from 2018 to 2019. It was comparing In this study, vitamin D levels were compared between patients with JIA and healthy children, and. This included an analysis of the relationship between vitamin D levels and relevant endogenous (age, sex, and clinical features of JIA), exogenous (region of residence, duration of sunshine, season of the year, vitamin D intake from food, and cholecalciferol intake), and geneticsgenetic (*VDR* gene variants) factors was analyzed. The study design is presented in Table 1.

**Table 1:** Design of our multicenter study evaluating the association between endogenous, exogenous, and genetic factors and vitamin D deficiency among children and adolescents with JIA.

**Groups**	**Clinical indicators, endogenous/exogenous factors**	**Genetic factors**
Healthy (*n* = 277) JIA (*n* = 150)	Age Sex Anthropometric indicators: Height (cm), Body weight (kg), BMI (kg/m^2^) Evaluation of vitamin D coming from diet Evaluation of the preventive dose of vitamin D Clinical manifestations Process activity Therapy Season of the year Region of residence	*VDR:* *FokI* (rs2228570, NM_000376.2:c.2T>C) *TaqI* (rs731236, NM_000376.2: c.1056T>C) *BsmI* (rs1544410, NM_000376.2: c.1024+283G>A)

*BMI, body mass index; JIA, juvenile idiopathic arthritis*.

The study was approved by the Ethics Committee of the Research Center for Medical Genetics (Protocol, “Algorithm for diagnosis and correction of vitamin D deficiency in children of the Russian Federation;” No. 9; dated December 8, 2017). Consent for participation and publication of the data was obtained from parents and/or participants, as appropriate based on the age of the participant.

### Characteristics of the Study Sample

The study sample comprised 150 patients with different variants of JIA (JIA group) and 277 healthy children (control group). Participants from three regions of the Russian Federation were included: the Moscow region (Sechenov University, Moscow; Research Clinical Institute for Childhood of the Moscow region, Moscow), the Krasnoyarsk territory (“Krasnoyarsk Interdistrict Clinical Hospital No. 20 named after I.S. Berzon,” Krasnoyarsk), and the Stavropol territory (Regional Children's Clinical Hospital of Stavropol). Patients with JIA at the time of the study were receiving inpatient treatment and had varying degrees of disease activity. The healthy control group comprised schoolchildren without chronic diseases whose parents agreed to participation of their child in the study.

The inclusion criteria for the JIA group were as follows: age, 1–18 years; diagnosis of JIA based on the International League of Associations of Rheumatology criteria (19); absence of other chronic diseases; and no history of acute respiratory infections in the 1 month prior to blood sampling for the study. All children in the JIA group underwent comprehensive clinical, laboratory, and instrumental examinations for assessment of their health status and disease activity. The therapy received was documented for inclusion in the analyses. The inclusion criteria for the control group were as follows: age, 1–18 years; absence of chronic diseases; and no history of acute respiratory infections in the 1 month prior to blood sampling for the study.

### Clinical, Laboratory, and Instrumental Assessments Performed for the JIA Group

Patients with JIA underwent comprehensive clinical, laboratory, and instrumental examinations in accordance with international recommendations. JIA activity was evaluated by the researchers according to the Juvenile Arthritis Disease Activity Score 10 (JADAS10). The cutoffs were as follows: ≤1 for inactive disease (ID); 1.1–2 for low disease activity (LDA) in oligoarthritis and 1.1–3.8 in polyarthritis; 2.1– 4.2 for moderate disease activity (MDA) in oligoarthritis and 3.9–10.5 in polyarthritis; > 4.2 for high disease activity (HAD) in oligoarthritis and >10.5 in polyarthritis (20, 21).

#### Measurement of Vitamin D Levels

The blood level of vitamin D was assessed by assaying its intermediate metabolite, calcidiol (25(OH)D). Enzyme immunoassays were performed using EUROIMMUN AG kits (Germany), with an EnSpire flatbed spectrofluorometer (PerkinElmer, Finland). The measurement range for this analysis was 4 ng/mL (10 nmol/L) to 150 ng/mL (375 nmol/L). Serum samples were obtained as part of a routine laboratory examination at regional centers; they were then frozen and stored at −20°C until analysis. Laboratory tests for all samples were performed simultaneously at the Research Center for Medical Genetics (Moscow). Vitamin D levels were measured four times in one calendar year, once in each season (Table 2).

**Table 2:** Number of blood samples used for the assessment of serum vitamin D levels in the JIA and control groups and for each season for each geographical region included in our study.

**Regions**	**Group**	**Samples (** ***n*** **)**				
		**Winter**	**Spring**	**Summer**	**Autumn**	**Total**
Moscow	JIA	22	4	23	17	66
	Healthy	54	11	20	39	124
Stavropol	JIA	21	16	22	21	80
	Healthy	29	25	28	68	150
Krasnoyarsk	JIA	16	9	31	16	72
	Healthy	28	27	34	28	117
Total	JIA	59	29	76	54	218
	Healthy	111	63	82	135	391

*Serum vitamin D levels were interpreted as per the 2011 recommendations of the International Society of Endocrinologists (20, 21): deficiency, calcidiol (25(OH)D) level <20 ng/mL; insufficiency, 20–29 ng/mL; and normal, 30–100 ng/mL*.

*JIA, juvenile idiopathic arthritis*.

#### Assessment of the Level of Insolation

The level of insolation was estimated for each region using data on the hours of sunshine obtained from the regional centers for hydrometeorology and environmental monitoring (Table 3). There were no significant differences in the overall days of sunlight between regions. The minimum insolation was recorded in winter (*p* < 0.01 compared with the insolation level for all other seasons) and the maximum in summer (*p* < 0.01 compared with the insolation level for all other seasons). There was no significant difference in the level of insolation between autumn and spring.

**Table 3:** Duration of sunshine (hours) for each season for each geographical region included in our study.

**Region/Territory**	**Winter**	**Spring**	**Summer**	**Autumn**	**Total**
Moscow	126.0	742.2	883.8	391.7	2,143.7
Krasnoyarsk	230.6	561.3	896.6	345.2	2,033.7
Stavropol	114.3	687.0	1,013.3	559.3	2,373.9

#### Assessment of Dietary Intake of Vitamin D

The dietary intake of vitamin D for the JIA group was calculated based on a 3-day menu questionnaire using the “Monitoring nutritional status, diet, and enzyme therapy” computer program (Certificate of state registration of the computer program: FIPS No. 2016 660762, dated 21.09.16).

#### DNA Analysis

Total DNA was isolated from the whole blood samples of patients in the JIA group using the standard phenol-chloroform extraction method. Polymorphic variants of xenobiotic biotransformation phase 1 genes were studied by amplification of the corresponding genomic regions by polymerase chain reaction, with analysis of polymorphic variants of the *VDR* gene performed by restriction analysis (PDRF). The polymorphisms *FokI, TaqI*, and *BsmI* of the *VDR* gene were studied.

#### Statistical Analysis

All analyses were performed using AtteStat (IBM SPSS Statistics 24). The Shapiro-Wilk test was used to determine the distribution type of data variables. As all distributions were non-normal, the data are presented as the median (Me) and 1st and 3rd quartiles [1Q and 2Q]. The non-parametric Mann-Whitney U test used to evaluate between-group comparisons for continuous variables and the Pearson's chi-squared test (χ^2^) and the exact Fisher's test for categorical data, as appropriate for the sample size: χ^2^ for variables with <30 samples and the Fisher's exact test for ≤4 samples. Spearman's (p) and Pearson's correlation coefficients were used to assess the relationships between indicators, as appropriate. Differences were considered statistically significant at *P* < 0.05.

## Results

A total of 218 blood samples were obtained from the JIA group and 391 from the control group. Over the study period, samples from 38 patients with JIA and 36 healthy controls were examined. In the JIA group, the proportion of girls was higher than that of boys (101; 67.3%), which is typical for the known sex distribution of the disease. For the control group, the sex ratio was close to equal (127 boys, 45.8%; 150 girls, 54.2%). The average age of participants in both the groups was 8.6 ± 0.2 years. The distribution of the type of JIA was as follows: systemic JIA, 12.0%; oligoarticular, 44.7%; polyarticular rheumatoid factor (RF)-negative, 32.7%; polyarticular RF-positive, 2.0%; enthesitis-related arthritis, 7.3%; psoriatic arthritis, 0.7%; and non-determined subtype, 0.7%. Active disease manifested in 97.3% of patients, with the remaining 2.7% being in the inactive phase (Table 4).

**Table 4:** Characteristics of patients in the JIA group.

**Characteristics**	**Total, *n* = 150**
Age of diagnosis, median [Q1; Q3], years	5.0 [2.8; 8.0]
Weight, median [Q1; Q3], kg	29.5 [19.0; 48.0]
BMI, median [Q1; Q3], kg/m^2^	16.6 [14.9; 19.5]
System, *n* (%)	18 (12.0%)
Oligoarticular, *n* (%)	67 (44.7%)
Polyarticular RF-negative, *n* (%)	49 (32.7%)
Polyarticular RF-positive, *n* (%)	3 (2.0%)
Enthesitis-related arthritis, *n* (%)	11 (7.3%)
Psoriatic arthritis, *n* (%)	1 (0.7%)
Undifferentiated, *n* (%)	1 (0.7%)
Non-active disease, *n* (%)	4 (2.7%)
Active disease, *n* (%)	146 (97.3%)
Uveitis, *n* (%)	25 (16.7%)
Positive ANF*, *n* (%)	23 (15.3%)
Treatment at the time of examination	
Methotrexate, *n* (%)	119 (79.3%)
Sulfasalazine, *n* (%)	37 (24.7%)
Biological preparations, *n* (%)	51 (34.0%)
Glucocorticoids orally and/or intravenously, *n* (%)	24 (16.0%)
NSAIDs, *n* (%)	50 (33.3%)
Cholecalciferol, *n* (%)	64 (42.7%)
Calcium preparations, *n* (%)	63 (42.0%)

*ANF, antinuclear factor; BMI, body mass index; JIA, juvenile idiopathic arthritis*.

### Vitamin D Status

**Figure 1:**
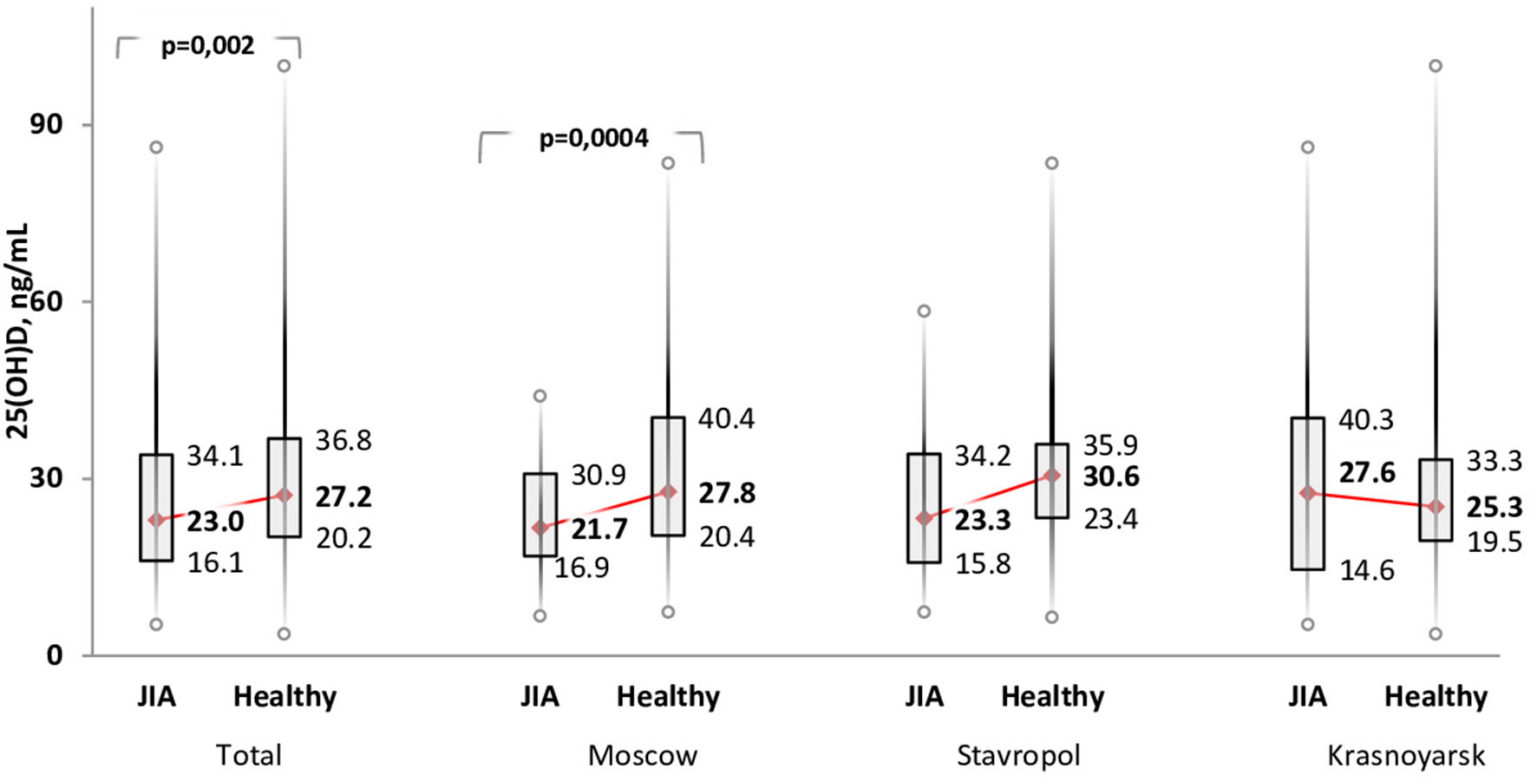
Comparison of the level of vitamin D (serum calcidiol, ng/mL) between the JIA and control group by geographical region included in the analysis.

The Me serum calcidiol level was lower in the JIA group (23.0 ng/mL) than in the control group (27.2 ng/mL; *P* = 0.002). The frequency of vitamin D (calcidiol) levels were as follows: <20 ng/mL, 57 (38.0%) in the JIA group and 68 (24.5%) in the control group (P=0.004); severe deficiency, 15 (10.0%) in the JIA group and one (0.4%) in the control group (*P* > 0.05); 21–29 ng/mL, 42 (28.0%) in the JIA group and 88 (31.8%) in the control group (*P* = 0.42); and ≥30 ng/mL, 51 (34.0%) in the JIA group and 121 (43.7%) in the control group (*P* = 0.052). Analysis of calcidiol level by region of residence revealed significant differences between the JIA and control groups for participants in Moscow (*P* = 0.004) but not for those in Stavropol (*P* = 0.06) or Krasnoyarsk (*P* = 0.96; Figure 1). No significant differences were noted in the distribution of vitamin D status within the JIA and control groups among the different regional centers (Table 5A).

**Table 5A:** Distribution of calcidiol (25(OH)D) level within each group according to each geographical region.

**Groups**		**25(OH)D**							
		**Me [1Q; 3Q]**		** <20 ng/mL**		**21–29 ng/mL**		**>30 ng/mL**	
		**ng/mL**	***P*-value**	***n* (%)**	***P*-value**	***n* (%)**	***P***	***n* (%)**	***P*-value**
Moscow region (1)	JIA, *N* = 66	21.7 [16.9; 30.9]	**0.0004**	28 (42.4%)	**0.007**	20 (30.3%)	0.41	18 (27.3%)	**0.01**
	Healthy, *n* = 117	27.8 [20.4; 40.4]		27 (23.1%)		36 (30.8%)		54 (46.1%)	
Stavropol (2)	JIA, *n* = 37	23.3 [15.8; 34.2]	0.06	12 (32.4%)	0.22	12 (32.4%)	0.46	13 (35.1%)	0.08
	Healthy, *n* = 44	30.6 [23.4; 35.9]		9 (20.5%)		11 (25.0%)		24 (54.5%)	
Krasnoyarsk (3)	JIA, *n* = 47	27.6 [14.6; 40.3]	0.96	17 (36.2%)	0.28	10 (21.3%)	0.08	20 (42.6%)	0.52
	Healthy, *n* = 116	25.3 [19.5; 33.3]		32 (27.6%)		41 (35.3%)		43 (37.1%)	
JIA	P 1-2	0.39		0.32		0.82		0.40	
	P 2-3	0.51		0.88		0.10		0.49	
	P 1-3	0.16		0.50		0.29		0.09	
Healthy	P 1-2	0.09		0.43		0.46		0.16	
	P 2-3	0.07		0.36		0.21		0.05	
	P 1-3	0.67		0.72		0.47		0.34	
Criterion	Mann-Whitney U		Pearson's (χ2)						

*The between-group P-value is shown for each region/territory. The between-region differences are reported using either the Mann-Whitney U for serum levels and Pearson's χ^2^ for the count in each category of calcidiol range*.

*JIA, juvenile idiopathic arthritis*.

*P 1-2 – comparison of Moscow region and Stavropol territory*.

*P 2-3 – comparison of Stavropol and Krasnoyarsk territories*.

*P 2-3 – comparison of Moscow region and Krasnoyarsk territory. Bold values are statistically significant (P < 0.05)*.

Calcidiol levels were significantly lower in the JIA group than in the control group across the age range of the study sample. The calcidiol level was negatively correlated with age in both the JIA and control groups, with this correlation being low for the JIA group (r = −0.18; *P* = 0.03) and moderate for the control group (r = −0.25; *P* < 0.0001). In the JIA group, the age of patients with vitamin D deficiency (Me, 9.7 [6.5; 13.1] years) was comparable to the age of patients with vitamin D insufficiency (Me, 9.3 [5.3; 13.1] years) and satisfactory vitamin D levels (Me, 8.4 [5.0; 11.0] years) (*P* > 0.05 for all comparisons). There was no between-sex difference in calcidiol levels; therefore, this criterion was not considered in further analyses.

Body weight was significantly higher in children with hypovitaminosis D, defined by calcidiol level of <30 ng/mL, (Me, 33.0 [19.5; 48.0] kg), than in those with satisfactory vitamin D levels, defined by calcidiol level of ≥30 ng/mL, (Me, 23.2 [18.0; 34.0] kg; *P* = 0.048), with similar findings noted for body mass index (BMI): for calcidiol level of <30 ng/mL, BMI was 17.3 [15.5; 19.6] kg/m^2^, and for calcidiol level of ≥30 ng/mL, BMI was 15.7 [14.5; 17.8] kg/m^2^ (*P* = 0.02).

### Forms of JIA, Disease Activity, and Vitamin D Status

The median serum calcidiol levels for the different forms of JIA were as follows: systemic, 21.3 [15.4; 30.3] ng/mL; oligoarticular, 25.8 [19.4; 37.6] ng/mL; polyarticular, 21.7 [15.8; 31.6] ng/mL; enteritis-associated arthritis, 20.2 [9.5; 22.0] ng/mL; psoriatic arthritis, 19.8 ng/mL; and undifferentiated arthritis, 21.9 ng/mL. By contrast, no significant differences were noted in vitamin D availability across the different forms of JIA. The distribution of vitamin D provision in patients with JIA, depending on the form of the disease, is presented in Table 5B. Between-group differences were compared with the oligoarticular form JIA group as the vitamin D provision status is best known to affect this group compared with other variant forms of JIA. The disease activity score, measured using the JADAS10, for the different vitamin D provision status groups was as follows: deficiency, 17.0 [10.5; 21.1] points; insufficiency, 16.5 [12.0; 20.0] points; and satisfactory, 11 [9.0; 16.0] points. The JIA disease activity score was significantly higher in the vitamin D deficiency group than in the normal vitamin D provision group (*P* = 0.03). There were no significant differences in the JADAS10 score between the vitamin D deficiency and satisfactory provision groups (*p* = 0.06) or between the vitamin D deficiency and insufficiency groups (*P* = 0.6). A significant negative correlation of average strength was noted between the disease activity score and the 25(OH)D level (r = −0.25, *P* = 0.002). Reverse analysis of 25(OH)D levels did not reveal significant differences in patients according to different levels of JIA activity. The vitamin D level was 27.4 (20.2; 29.1) ng/mL in patients with low JIA activity, 27.7 (18.5; 39.4) ng/mL in patients with average JIA activity, and 22.1 (15.4; 33.5) ng/mL in those with high JIA activity (*P* = 0.13 comparing patients with low and average JIA activity versus those with high JIA activity).

**Table 5B:** The distribution of vitamin D provision for patients with JIA depending on the form of the disease.

**Characteristics**	**25(OH)D**					^**^ *P*
	**Me [Q1; Q3]**	^*^ *P*	**<20 ng/mL *n* (%)**	**21–29 ng/mL *n* (%)**	**>30 ng/mL, *n* (%)**	
			**1**	**2**	**3**	
Oligoarticular, *n* = 67	25,8 [19,4; 37,6]	-	21 (31,3%)	17 (25,4%)	29 (43,3%)	-
Polyarticular RF-negative, *n* = 49	21,7 [15,8; 31,6]	0,14	20 (40,8%)	14 (28,6%)	15 (30,6%)	p_1_ = 0,292 p_2_ = 0,701 p_3_ = 0,165
Polyarticular RF-positive, *n* = 3			2 (66,7%)	1 (33,3%)	-	p_1_ = 0,203 p_2_ = 0,758
Systemic, *n* = 18	21,3 [15,4; 30,3]	0,24	8 (44,4%)	5 (27,8%)	5 (27,8%)	p_1_ = 0,298 p_2_ = 0,837 p_3_ = 0,234
Enthesitis-related arthritis, *n* = 11	20,2 [9,5; 22,0]	0,06	5 (45,5%)	4 (36,4%)	2 (18,2%)	p_1_ = 0,358 p_2_ = 0, 447 p_3_ = 0,115
Psoriatic, *n* = 1	19,8	-	1 (100%)	-	-	-
Undifferentiated, *n* =1	21,9	-	-	1 (100%)	-	-

* *Level of significant differences between vitamin D provision for the oligoarticular JIA group compared with other forms of JIA using the Mann-Whitney test*.

** *Level of significant differences between vitamin D provision for the oligoarticular JIA group compared with other forms of JIA using the Pearson criterion (χ2)*.

*JIA, juvenile idiopathic arthritis*.

### Seasonal Differences in Vitamin D Availability

**Figure 2:**
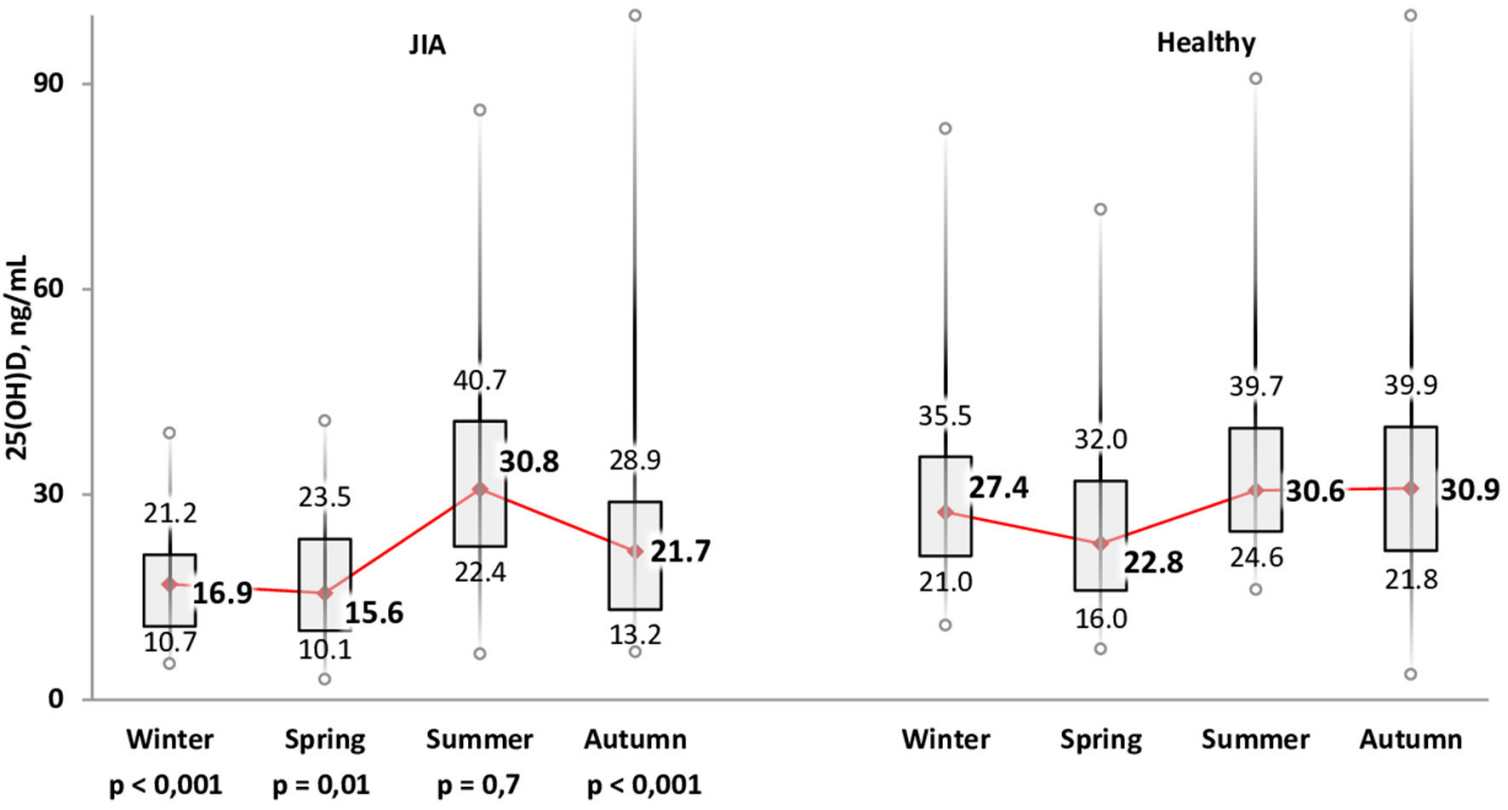
Calcidiol (25(OH)D) level (ng/mL) for the JIA and control groups for each season.

**Figure 3:**
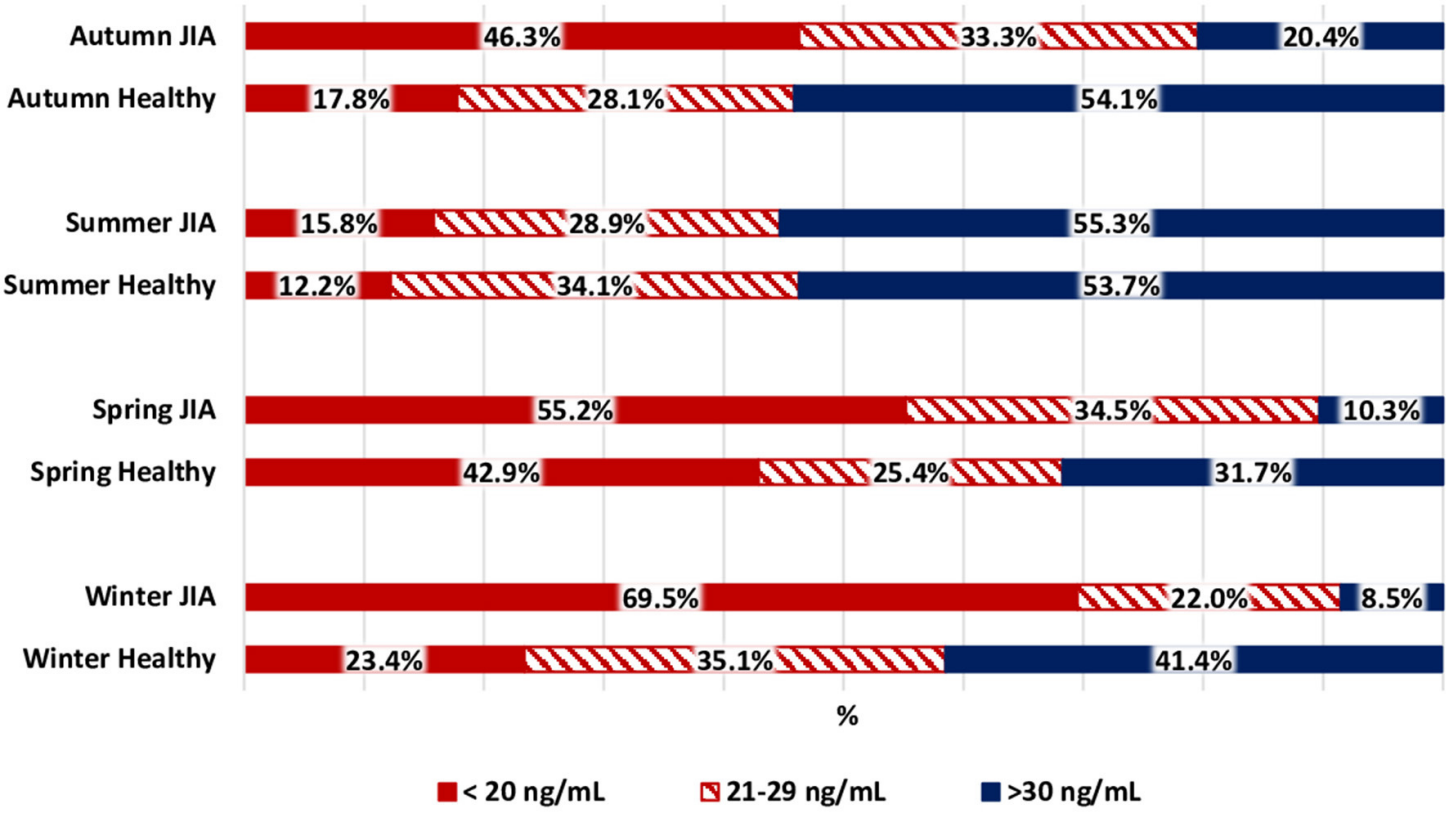
Comparison of the distribution of vitamin D status among the JIA and control groups for each season.

The variation in calcidiol levels by season is shown for both the JIA and control groups in Figure 2. The highest level was recorded in summer (Me, 30.8 [22.4; 40.7] ng/mL) and the lowest in winter (Me, 16.9 [10.7; 21.2] ng/ml) and spring (Me, 15.6 [10.1; 23.5] ng/mL). In winter, spring, and autumn, vitamin D levels were significantly lower in the JIA than in the control group (*P* < 0.001, *P* = 0.01, and *P* < 0.001, respectively), with no between-group difference noted in summer (Figure 3). The statistical significance of these between-season comparisons, as a function of the level of vitamin D availability, is shown in Table 6.

**Table 6:** Between-season comparison of vitamin D levels.

		**Serum calcidiol level**		
***P*-value**	**Between-season comparison**	** <20 ng/mL**	**21–29 ng/mL**	**>30 ng/mL**
	Winter-Spring	0.19	0.21	0.78
	Winter-Summer	**<0.001**	0.36	**<0.001**
	Winter-Autumn	**0.01**	0.18	0.07
	Spring-Summer	**<0.001**	0.58	**<0.001**
	Spring-Autumn	0.44	0.43	0.25
	Summer-Autumn	**<0.001**	0.59	**<0.001**

*P-value calculated using the Pearson's χ2 test. Bold values are statistically significant (P < 0.05)*.

The frequency of vitamin D deficiency in the range of 21–29 ng/mL was 22.0–34.5% over the full year. In summer, during which vitamin D level in the JIA group is maximum, the frequency of satisfactory vitamin D levels reached 55.3%, which is significantly higher than the frequency in winter, spring, and autumn. During the period of minimal insolation, from September to May, the frequency of vitamin D deficiency was higher in winter than in autumn. The correlation between days of sunlight and calcidiol levels was moderate over the full year (r = 0.36; *P* < 0.0001.) The analysis of vitamin D availability in the JIA group as a function of various basic therapies used is presented in Table 7. No significant effect of therapy used was noted on the frequency of vitamin D status (deficiency, insufficiency, and normal).

**Table 7:** Distribution of vitamin D status among patients in the JIA group as a function of the therapy used.

**Therapy**	**25(OH)D**, ***n*** **(%)**			***P*-value**
	** <20 ng/mL**	**21–29 ng/mL**	**>30 ng/mL**	
	**1**	**2**	**3**	
Methotrexate, *n* = 119	46 (38.7%)	32 (25.9%)	41 (34.5%)	p_1_ = 0.75 p_2_ = 0.56 p_3_ =0.82
Without methotrexate, *n* = 31	11 (35.5%)	10 (32.3%)	10 (32.3%)	
Sulfasalazine, *n* = 37	12 (32.4%)	12 (32.4%)	13 (35.1%)	p_1_ = 0.42 p_2_ = 0.49 p_3_ =0.87
Without sulfasalazine, *n* = 113	45 (39.8%)	30 (26.5%)	38 (33.6%)	
Biological preparations, *n* = 51	22 (43.1%)	16 (31.4%)	13 (25.5%)	p_1_ = 0.36 p_2_ = 0.51 p_3_ =0.12
Without biological preparations, *n* = 99	35 (35.4%)	26 (26.3%)	38 (38.4%)	
Systemic glucocorticoids, *n* = 24	9 (37.5%)	8 (33.3%)	7 (29.2%)	p_1_ = 0.98 p_2_ = 0.53 p_3_ =0.59
Without systemic glucocorticoids, *n* = 126	48 (38.1%)	34 (27.0%)	44 (34.9%)	
NSAIP, *n* = 50	21 (42.0%)	15 (30.0%)	14 (28.0%)	p_1_ = 0.75 p_2_ = 0.56 p_3_ =0.82
Without NSAIP, *n* = 100	36 (36.0%)	27 (27.0%)	37 (37.0%)	
Total vitamin D intake (vitamin D preparations and vitamin D together with calcium), *n* = 89	29 (32.6%)	26 (29.2%)	34 (38.2%)	^*^p_1_ = 0.10 ^*^p_2_ = 0.69 ^*^p_3_ =0.19
Vitamin D preparations, *n* = 26	6 (23.1%)	6 (23.1%)	14 (53.8%)	^*^p_**1**_ **=** 0.04 ^*^p_2_ = 0.76 ^*^p_**3**_ **=0.02**
Vitamin D together with calcium, *n* = 63	23 (36.5%)	20 (31.7%)	20 (31.7%)	^*^p_1_ = 0.29 ^*^p_2_ = 0.50 ^*^p_3_ =0.69
Without vitamin D, *n* = 61	28 (45.9%)	16 (26.2%)	17 (27.9%)	-

* *Compared to patients not receiving vitamin D and calcium supplementation*.

*JIA, juvenile idiopathic arthritis. Bold values are statistically significant (P < 0.05)*.

### Effect of Dietary Intake on Vitamin D Availability

Among children in the JIA group, dietary intake of vitamin D was only 6–13% of the recommended norm (10 mcg per day) (10), regardless of the vitamin D status (*P* > 0.05). Of note, there was no correlation between the dietary intake of vitamin D and the serum calcidiol level (*P* > 0.05).

### Effect of Prophylactic Doses of Cholecalciferol on Vitamin D Availability

An extremely important stage of the analysis was the comparison of calcidiol levels between the JIA and control groups among those who received a daily dose vitamin D supplementation (cholecalciferol). Vitamin D supplementation was provided in 89 (59.3%) of patients in the JIA group (Me dose, 900 [400; 1,000] IU/day) and 109 (39.4%) in the control group (Me dose, 1,000 [500; 1,500] IU/day), the dose being higher for the control group than for the JIA group (*P* = 0.002). For the JIA group, the serum calcidiol levels were higher among those receiving cholecalciferol supplementation (Me, 25.3 [19.2; 36.0] ng/mL) than among those who did not receive supplementation (Me, 21.3 [12.6; 29.8] ng/mL; *P* = 0.013). Similarly, in the control group, vitamin D supplementation was associated with higher serum calcidiol levels (with supplementation, Me, 34.5 [25.0; 44.6] ng/mL, and without supplementation, Me, 24.4 [18.7; 31.6] ng/mL; *P* < 0.0001). Among those who did not receive vitamin D supplementation, controlling for a background of pharmacoprophylaxis for vitamin D deficiency among patients in the JIA group, serum calcidiol levels were significantly higher in the control group than in the JIA group (*P* < 0.001).

**Figure 4:**
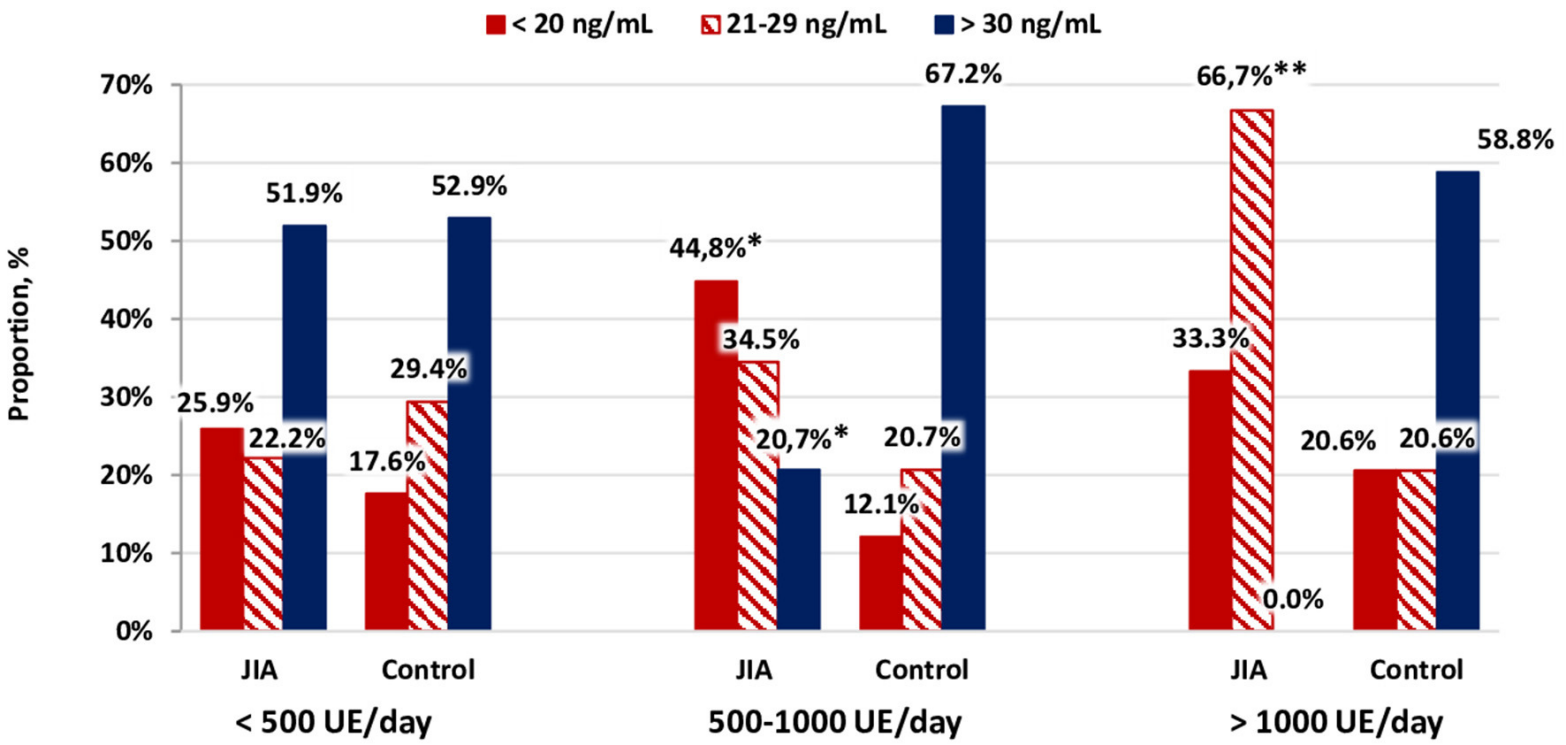
Distribution of vitamin D status as a function of the dose of cholecalciferol supplementation.*p < 0.001, comparison between the JIA and control group, calculated using Pearson's χ2. **p < 0.05, comparison between the JIA and control group, calculated using Fisher's exact test.

Figure 4 shows that the supplementation of cholecalciferol at a dose of 500–1,000 IU/day resulted in a 3.5 times higher frequency of vitamin D deficiency in the JIA group than in the control group, with no between-group difference noted when the cholecalciferol dose in patients with JIA was >1,000 IU/day. Of note, cholecalciferol deficiency was detected among patients with JIA three times more often compared with that noted among individuals in the control group. Our findings indicate that vitamin D supplementation among healthy children, using the dose recommended by the National Program “Vitamin D deficiency in children and adolescents of the Russian Federation: actual approaches to correction” (4), is effective and does not lead to excessively increased serum calcidiol levels. Patients with JIA require higher therapeutic and prophylactic doses of cholecalciferol to achieve a serum calcidiol level of ≥30 ng/mL compared with that in healthy children.

### Influence of Genotypes of Polymorphisms of the *VDR* Gene on Serum Levels of Calcidiol

Among 106 patients with JIA, genotypes were established according to *TaqI* and *ApaI* polymorphisms in 71, *FokI* (BstF5) *VDR* (c.152T>C) in 70, and *Pct I* (BsmI) *VDR* (c.1174+283G>A) in 59. Among them, only the 97 patients who did not receive vitamin D supplementation were included so that the average vitamin D level was determined as a function of the genotypes of polymorphisms of the *VDR* gene in winter and summer. There was no significant association between the genotype of *VDR* gene polymorphisms and the average serum level of calcidiol, either in summer or winter (Table 8).

**Table 8:** Serum calcidiol level among patients with JIA who did not receive vitamin D supplementation, as a function of the genotypes of polymorphisms of the *VDR* gene, in winter and summer.

***VDR*** **polymorphism**		***N***	**25(OH)D, ng/mL**		***P*-value**	***P*-value** **Summer-Winter**
		**M ±SD**	**Me [1Q; 3Q]**			
c.1206T>C(A>G) TaqI Summer	TT (1)	18	32.0 ± 11.6	30.6 [23.4; 42.6]	p_1−2_ = 0.222 p_1−3_ = 0.113 p_2−3_ = 0.399	**p**_**1−4**_ **=** **0.001** **p**_**2−5**_ **=** **0.000** p_3−6_ = 0.288
	TC (2)	30	27.4 ± 13.0	25.6 [18.5; 37.0]		
	CC (3)	4	21.6 ± 9.9	21.2 [14.8; 28.5]		
	Total	52	28.6 ± 12.4	27.7 [19.8; 39.3]		
c.1206T>C(A>G) TaqI Winter	TT (4)	13	17.7 ± 10.2	15.1 [13.0; 19.8]	p_4−5_ = 0.197 p_4−6_ = 0.483 p_5−6_ = 0.769	
	TC (5)	16	13.7 ± 6.2	11.7 [9.3; 15.8]		
	CC (6)	2	12.4 ± 3.2	12.4 [10.1; 14.6]		
	Total	31	15.3 ± 8.1	13.2 [10.1; 17.0]		
c.152T>C FokI Summer	TT (1)	7	24.0 ± 13.9	25.1 [6.7; 38.0]	p_1−2_ = 0.593 p_1−3_ = 0.111 p_2−3_ = 0.128	p_1−4_ = 0.058 **p**_**2−5**_ **=** **0.023** **p**_**3−6**_ **=** **0.000**
	TC (2)	23	26.9 ± 12.5	26.9 [14.1; 37.0]		
	CC (3)	20	32.5 ± 11.2	30.0 [23.4; 42.6]		
	Total	50	29.2 ± 12.2	28.6 [20.8; 40.5]		
c.152T>C FokI Winter	TT (4)	7	12.5 ± 4.2	14.6 [8.1; 15.9]	p_4−5_ = 0.232 p_4−6_ = 0.557 p_5−6_ = 0.451	
	TC (5)	13	17.4 ± 9.9	13.2 [12.1; 19.8]		
	CC (6)	10	14.5 ± 7.9	10.9 [8.6; 22.9]		
	Total	30	15.3 ± 8.2	13.1 [10.1; 17.0]		
c.1174+283G>A BsmlI Summer	AA (1)	3	25.5 ± 7.6	22.9 [19.5; 34.1]	p_1−2_ = 0.820 p_1−3_ = 0.193 p_2−3_ = 0.134	p_1−4_ = 0.113 **p**_**2−5**_ **=** **0.000** **p**_**3−6**_ **=** **0.001**
	GA (2)	23	27.4 ± 13.9	25.2 [16.1; 40.5]		
	GG (3)	14	33.9 ± 10.0	34.2 [26.9; 42.6]		
	Total	40	29.5 ± 12.5	28.6 [21.4; 40.7]		
c.1174+283G>A BsmlI Winter	AA (4)	2	12.4 ± 3.2	12.4 [10.1; 14.6]	p_4−5_ = 0.773 p_4−6_ = 0.474 p_5−6_ = 0.167	
	GA (5)	16	13.7 ± 6.1	11.7 [9.3; 15.5]		
	GG (6)	11	18.4 ± 11.0	16.6 [12.4; 24.4]		
	Total	29	15.4 ± 8.4	13.0 [10.1; 17.0]		
ApaI VDR (c.1175-49G>T) Summer	TT (1)	9	26.3 ± 11.6	25.1 [19.5; 33.7]	p_1−2_ = 0.547	**p**_**1−4**_ **=** **0.005** **p**_**2−5**_ **=** **0.000**
	TG (2)	43	29.0 ± 13.0	27.8 [20.1; 42.6]		
	GG (3)	0	-	-		
	Total	52	28.6 ± 12.4	27.7 [19.8; 39.3]		
ApaI VDR (c.1175-49G>T) Winter	TT (4)	6	13.2 ± 2.7	13.2 [10.7; 15.6]	p_4−5_ = 0.490	
	TG (5)	25	15.8 ± 8.9	13.2 [9.6; 19.8]		
	GG (6)	0	-	-		
	Total	31	15.3 ± 8.1	13.2 [10.1; 17.0]		

*M, mean; SD, standard deviation; Me, median; Q1-Q3, 1^st^ and 3^rd^ interquartile range; JIA, juvenile idiopathic arthritis*.

*Between-group differences for summer and winter for each polymorphism was using the Mann-Whitney U-test*.

*Groups 1,2,3: winter; groups 4,5,6: summer; and groups 7,8,9: winter + summer. Bold values are statistically significant (P < 0.05)*.

## Discussion

This study compared vitamin D availability, measured as the serum calcidiol levels, between patients with JIA and their healthy counterparts and found that vitamin D levels are low in children and adolescents across all three geographical regions of the Russian Federation, namely Moscow, Krasnoyarsk, and Stavropol, with vitamin D deficiency (serum calcidiol level, <20 ng/mL) being identified in 38.0% of participants in the JIA group and 24.5% in the control group. We identified severe deficiency in 10.0% of participants in the JIA group and 0.4% in the control group and satisfactory vitamin D availability in 34.0% of participants in the JIA group and 43.7% in the control group. Various factors can contribute to these between-group differences in vitamin D levels, including the presence of JIA as a chronic disease and the associated immobility and decreased time participating in outdoor activities. We noted that families with a child with JIA tend to live in large cities where highly qualified medical care is more accessible; however, the air quality is often poorer than that in rural regions. Moreover, patients with JIA often have an increased BMI and receive medications that can negatively impact vitamin D metabolism over prolonged periods of time. The tendency among children with JIA to have a higher BMI is clinically important as vitamin D is fat-soluble and can thus be deposited in adipose tissue, resulting in a lower calcidiol (25(OH)D) level in the blood (22). The results of an earlier meta-analysis confirmed the association between obesity in children, adolescents, and adults, regardless of age and geographical latitude, and low serum vitamin D levels (23).

Our data regarding the prevalence of vitamin D deficiency among healthy children in Russia are consistent with those of a previous study reporting a prevalence of vitamin D insufficiency in 30.1%, deficiency in 19.8%, severe deficiency in 1.6%, and normal levels in 45.5% of participants (24).

Insufficient vitamin D among children with JIA has been noted in various countries. Pelajo et al. (25) reported vitamin D deficiency (calcidiol level ≤19 ng/mL) in 13% of children with JIA and vitamin D insufficiency (calcidiol level, 20–29 ng/mL) in 42%, which included patients receiving vitamin D supplementation. Comak et al. (26) reported that the majority of children with JIA (72.3%) had vitamin D deficiency (calcidiol level, <20 ng/mL), with 19.1% having levels of 15–20 ng/mL and 53.2% having levels <15 ng/mL. Marini et al. (27) reported a prevalence of vitamin D deficiency and insufficiency of 80.6 and 3.9%, respectively, among patients with JIA, with no increase noted in parathyroid hormone levels. A meta-analysis of 19 studies up to 2011 confirmed the high prevalence of vitamin D deficiency among patients with JIA, with the lowest average levels of 25(OH)D and 1.25(OH)2D among patients with a systemic form of the disease (18). A meta-analysis including 38 studies of patients with JIA and juvenile rheumatoid arthritis revealed that the average 25(OH)D level in children was suboptimal (<75 nmol/L) in 32 (84.2%) of the studies (28).

In our study, we evaluated various factors that may affect vitamin D intake and synthesis. In selecting these factors, we considered that vitamin D synthesis begins in the skin when exposed to UV radiation of a certain wavelength. Due to a higher body surface area to volume ratio and higher rate of vitamin D synthesis, children require less intense solar exposure than adults (29). One of the most significant factors determining the intensity of UV exposure is the geographical latitude where an individual lives. Up to 1,000 IU of D3 can be synthesized per day in the skin under the influence of UV radiation under optimal conditions at the equator (30). In their study of children with JIA living in the equatorial region, Studart de Souza et al. (31) reported vitamin deficiency in only 8% of children, with serum 25(OH)D levels being comparable between patients with JIA and healthy peers and with no effect noted on the variance or severity of JIA. Another study reported hypovitaminosis D in 75% of children with JIA in Morocco (32). According to a meta-review by Finch et al. (28), the average serum vitamin D level among patients with JIA decreases as a function of geographical location away from the equator. These authors also noted a relationship between low vitamin D status and disease activity, which also corresponded to the north-south gradient. This may be associated with the significant decrease in UV photons reaching the earth's surface above the 37° north latitude (up to 80–100% depending on the parallel) in winter from November to February (33). In territories above the 33° north latitude, the UV intensity is insufficient for vitamin D synthesis throughout the year (34–36). Indeed, in Central Europe and North America, considering the geographical location of the territory and the duration of outdoor activities, vitamin D synthesis averages at 500–600 IU/day in summer, with little to no synthesis in winter (37, 38). Even in Italy, children are unable to synthesize vitamin D in late autumn, winter, and early spring, despite the adequate insolation available (39). During these periods of low or absent vitamin D synthesis, the vitamin D status is maintained by endogenous accumulation from previous seasons and exogenous supplementation.

As the entire territory of Russia is located above the 41° north latitude, with a significant area above the 50° north latitude, UV is insufficient for vitamin D synthesis. This explains the absence of differences in vitamin D levels across the three geographical regions included in our study, all of which were located 10° apart from each other. The autoinflammatory process and severity of the disease did not influence the impact of geographical locations and, thus, period of insolation. Overall, considering the climatic and geographic conditions of Russia, appropriate dietary intake and supplementation of vitamin D are necessary to compensate for vitamin D deficiency.

Herein, all the children and adolescents with JIA were receiving inpatient treatment at the time of this study. Patients with a high degree of JIA activity needed hospitalization, and the indicators of the JADAS10 were high. Using the classification proposed by Consolaro et al. (20), low JIA activity was detected in 4 (2.7%), average JIA activity in 36 (24%), and high JIA activity in 110 (73.3%) patients, with high activity being a clear majority. Consolaro et al. noted that clinical JADAS correlates with JADAS3-10, JADAS-27, and JADAS-71 (20, 21). At the hospital, the determination of erythrocyte sedimentation rate (ESR) is an affordable and routine diagnostic method. Therefore, we chose the standard JADAS10 scale, which includes ESR as a marker of the acute phase of inflammation. In our study, the JADAS10 level was significantly higher in patients with JIA and vitamin D deficiency than in patients with 25(OH)D levels of >30 ng/mL (*P* = 0.03). JIA activity also correlated negatively with serum vitamin D levels (r = −0.25, *P* = 0.002). This could simply indicate that vitamin D level is associated with the amount of sunlight exposure in patients. It is impossible to completely refute the hypothesis that vitamin D deficiency itself is a predictor of a more active course of JIA; however, this remains to be studied in the future.

Vitamin D is positively associated with physical activity (40). In a study by van den Heuvel (41), the authors speculated whether physical activity influenced vitamin D due to changes in hormone levels. In addition, indoor muscle-building exercise is associated with increased levels of vitamin D (42). One explanation for this may be that vitamin D can be stored in muscles (43). A more recent study revealed a positive relationship between vitamin D and cardiorespiratory fitness measured by maximum oxygen consumption (VO2 max) in a middle-aged US population (44). However, patients with more severe disease activity are less mobile and may be restricted to spend time outdoors. Fatigue is a prevalent distressing symptom in children and adolescents with pediatric rheumatic conditions (45) and can have a significant impact on the well-being and participation in the daily life of the patient (46–48).

D2 (ergocalciferol) and D3 (cholecalciferol) can enter the body *via* an individual's diet, with ocean fish fat, animal liver fat, and marine egg yolks being primary sources (49). However, an adult resident of Central Europe receives only 80–120 IU of vitamin D per day on average from dietary intake (50). Similarly, the traditional Russian diet of children contains foods with a very small amount of vitamin D; therefore, dietary intake cannot be considered as a significant source of vitamin D. The only exceptions are in the northern regions of Russia, where oily fish, caviar, and fat from marine animals are abundant in the daily diet. These regions were not included in our study. Children with JIA in our study achieved only 6–13% of the physiological norm of daily dietary intake of vitamin D, which might explain the absence of a correlation between the dietary intake of vitamin D and serum calcidiol levels. Taken together, our study confirms the need for vitamin D supplementation in children with JIA as well as their healthy counterparts to achieve adequate levels of calcidiol. The question of the dose of vitamin D supplementation (cholecalciferol) necessary for the correction of vitamin D deficiency is being actively discussed, with varying opinions being presented.

Our data clearly demonstrate that the prophylactic doses of cholecalciferol do not currently meet the needs of children in the geographical regions included in our study. In patients with JIA who received cholecalciferol supplementation, the calcidiol level increased to 25.3 [Me] ng/mL, which was significantly higher than the levels in children who did not receive pharmacological support for vitamin D deficiency (21.3 [Me] ng/mL; between-group P=0.013). Similarly, in the control group, cholecalciferol supplementation significantly increased the serum calcidiol levels. With a cholecalciferol dose of 500–1,000 IU/day, the frequency of vitamin D deficiency among patients in the JIA group was 3.5 times higher than that in the control group. Importantly, with a dose >1,000 IU/day, the difference in the frequency of deficiency was not significant between the two groups, although vitamin D deficiency was still detected three times more frequently in the JIA group compared with that noted in the control group.

It is obvious that patients with JIA require elevated therapeutic and prophylactic doses of cholecalciferol to reach a normal 25(OH)D level of ≥30 ng/mL compared with that noted in healthy children. At present, no standard guidelines regarding appropriate vitamin D supplementation have been established. It has been shown that vitamin D supplementation at a dose of 1,000 IU/day for weeks/months resulted in a slow increase in 25(OH)D level at 15–25 nmol/L (51, 52). Recommendations from various authors presented in a recent review by Saggese et al. (53) indicate a dose of 2,000–5,000 IU of cholecalciferol per day to 50,000–60,000 IU per week to correct vitamin D deficiency in children and adolescents. The recommended duration of supplementation also varies widely from 6–8 to 12–16 weeks. Our study confirmed the need for higher preventive doses of cholecalciferol in patients with JIA. Concurrently, the usual daily dose was reported to be insufficient for patients receiving intensive therapy and should be increased to quickly improve the vitamin D status (54).

The effect of drug therapy on vitamin D status warrants dedicated attention. We do know whether 1,25(OH)2D increases the transcription of genes encoding several enzymes that affect the bioavailability and metabolism of drugs. Almost half of these drugs are metabolized by CYP3A4, and the remaining drugs have a stimulatory or an inhibitory effect on the activity of this enzyme. CYP3A4 is active in enterocytes and hepatocytes, indicating a possible interaction between oral medications and preparations of vitamin D supplementation. Moreover, impaired absorption of fat-soluble vitamin D is inevitable when taking medications that suppress fat absorption or when following a diet with its restriction (55).

It has recently been established that glucocorticosteroids (GCS) are capable of binding as ligands to the nuclear pregnane-X receptor, which regulates the expression of CYP450 genes that play a key role in the metabolism of xenobiotics and drugs. The activation of pregnane-X receptor, similar to that of *VDR*, affects the expression of 24-hydroxylase genes, which enhances the degradation of 25(OH)D and 1,25(OH)2D, accelerating the catabolism of vitamin D and leading to its deficiency (56). In patients receiving GCS, the risk of vitamin D deficiency is two times higher than that in the general population (57). Sulfasalazine reportedly increases the mRNA levels of endogenous 1α-hydroxylase *in vitro* (56). Experimental models have shown that cyclosporine inhibits CYP27A1 and reduces the expression of *VDR* and CYP24 and can probably change the 25(OH)D level. Tacrolimus is metabolized by CYP3A4 and CYP3A5; therefore, its level may also be associated with a change in 25(OH)D level. However, in clinical studies, noticeable effect of these drugs has been reported, and the genotypes of the studied polymorphisms of CYP2C9 (1075A>C; I359L), CYP2C19 (636G>A; W212X), and CYP3A4 (c.1334T>C; M445T) were not noted at the level of vitamin D (55). The results of our study did not reveal significant differences in the frequency of vitamin D deficiency, vitamin D insufficiency, and normal vitamin D in patients with JIA who received and did not receive sulfasalazine or methotrexate, which does not allow us to assess their possible effect on vitamin D metabolism. We did not identify an association between the vitamin D status and systemic GCS therapy. This is probably due to the fact that mainly low doses of GCS are currently used; when their effectiveness is insufficient, biologics are used. According to the literature, the effect of biologics (golimumab, adalimumab, and rituximab) on vitamin D levels in adults with rheumatoid arthritis has not been noted (56), which is consistent with our data.

In our study, calcidiol levels did not depend on the genetic variants of *FokI, TaqI*, and *BsmI VDR*. Several studies have demonstrated similar results, with no difference in 25(OH)D levels in the blood serum of female carriers of the rs1544410 (*BsmI*), rs7975232 (*ApaI*), rs731236 (*TaqI*), and rs2228570 (*FokI)* variants of the *VDR* gene (58). Another study found no association between serum vitamin D levels and *TaqI* variant polymorphisms of the *VDR* gene in an elderly population in Brazil (59). At the same time, it was shown that *SNP ApaI* (*T*>*G*) is associated with 25(OH)D levels in both men and women and that 25(OH)D levels were significantly lower in carriers of the *TT* genotype than in carriers of the *GG* genotype (60). The presence of the *AA* genotype of a polymorphic variant of *BsmI* is associated with a 1.84-fold increase in the risk of developing idiopathic arthritis (IA), with the presence of the A allele in the genotype being associated with a 1.34-fold increase in the probability of developing IA among patients without osteoarticular pathology (61). Kostik et al. (62) reported that the *BB BsmI* genotype of the polymorphic marker of the *VDR* gene is associated with high disease activity, which can be considered as a marker of an unfavorable prognosis in boys with JIA. In addition, there is evidence to consider vitamin D as a risk factor for the development of JIA. For example, *FokI, BsmI*, and *TaqI* polymorphisms of the *VDR* gene are associated with an increased frequency of JIA in different ethnic populations (63). In a recent study, the expression of vitamin D and *VDR* was significantly lower in patients with JIA than in those in the control group, with patients with low expression of the *VDR* gene having significantly higher disease activity and disability, probably in addition to the low vitamin D levels. Therefore, low *VDR* expression is associated with the inflammatory process and plays a potential role in the pathogenesis and prognosis of juvenile rheumatoid arthritis (64).

### Limitations

This study has some limitations. Cross-sectional levels of vitamin D were obtained in each season, while serial levels for each participant were not examined across the four seasons of the year. The inclusion of new patients allowed us to evaluate the overall prevention of vitamin D deficiency among children across the four seasons of the year. However, we used the duration of sunshine in each of the four geographic regions included in our study and not the individual exposure to sun. Therefore, this study determined the duration of sunshine in the regions and not children's exposure to the sun.

In conclusion, our study demonstrated the influence of exogenous and endogenous factors, including genetic factors, on vitamin D availability.

## Data Availability Statement

The original contributions presented in the study are included in the article/supplementary material, further inquiries can be directed to the corresponding author.

## Ethics Statement

The studies involving human participants were reviewed and approved by Ethics Committee of the Research Center for Medical Genetics. Written informed consent to participate in this study was provided by the participants' legal guardian/next of kin.

## Author Contributions

EK, NI, and LK performed the conceptualization and provided the proof outline for the research. EK contributed to the original idea. EK, NI, LK, NP, and NO supervised the research. YK, AZ, EZ, VK, SD, ES, LB, YM, TD, and NP all carried out the research. EL wrote the manuscript with support from NP, SD, EZ, and YK. All authors discussed the results and contributed to the final manuscript and contributed to the article and approved the submitted version.

## Funding

This work was performed in adherence to the state guidelines for scientific research: Patterns and mechanisms of bone mineral density reduction in healthy children and in various models of inflammation (microbial-inflammatory, allergic, metabolic and autoimmune). Improving prevention and therapy in real clinical practice (No. 121122200169-1).

## Conflict of Interest

The authors declare that the research was conducted in the absence of any commercial or financial relationships that could be construed as a potential conflict ofinterest.

## Publisher's Note

All claims expressed in this article are solely those of the authors and do not necessarily represent those of their affiliated organizations, or those of the publisher, the editors and the reviewers. Any product that may be evaluated in this article, or claim that may be made by its manufacturer, is not guaranteed or endorsed by the publisher.
